# Recording of Alcohol Use Disorder in Electronic Health Records: Developing a Recommended Codelist for Research

**DOI:** 10.2147/CLEP.S477778

**Published:** 2024-10-04

**Authors:** Sarah Cook, David Osborn, Arti Maini, Ravi Parekh, Shamini Gnani, Thomas Beaney, Ana Luisa Neves, Sonia Saxena, Jennifer K Quint

**Affiliations:** 1 School of Public Health, Imperial College London, London, UK; 2 Division of Psychiatry, University College London, London, UK; 3 Camden and Islington NHS Foundation Trust, London, UK

**Keywords:** alcohol use disorder, electronic health records, primary care, clinical practice research datalink

## Abstract

**Purpose:**

Electronic health records (EHR) are valuable resources for health research; however, their use is challenging. A validated alcohol use disorder (AUD) codelist for UK primary care is needed to improve population-based research in this patient group. We aimed to develop an AUD codelist for use in the Clinical Practice Research Datalink (CPRD) Aurum database, a UK EHR primary-care database.

**Methods:**

The CPRD code browser was searched using keywords related to alcohol use using a previously developed search strategy. The resulting codes were categorised as AUD if they were: a) diagnostic of AUD, b) indicated alcohol withdrawal, or c) indicated chronic alcohol-related harm (physical or mental). Codes related to alcohol use but not used to define AUD were also classified into relevant categories (alcohol status, acute harm, and alcohol screening). All codes were categorised independently by at least two reviewers (one person reviewed all codes and five reviewers (all practising GPs) each reviewed a subset of codes (100–200 codes each). Disagreements in categorisation were discussed by at least three coders and a consensus was reached. The reliability of categorisation was assessed using kappa statistics.

**Results:**

In total, 556 potential codes related to alcohol use were identified. The Kappa for reliability between coders was moderate for both AUD (0.72) and across all categories (0.62), with substantial variability between coders (AUD: 0.33–0.97; all categories 0.36–0.74). In the final codelist, 138 codes were included as indicating AUD: 38 codes identified which indicated diagnosis of AUD, 14 indicating withdrawal plus 85 codes indicating chronic alcohol-related harm (41 physical health and 44 mental health).

**Conclusion:**

Many codes are used in primary care to record alcohol use and associated harms, and there is substantial variability in how clinicians categorise them. While future work formally validating the codelist against gold standard clinical reviews and qualitative work with General Practitioners is needed for a deeper understanding of coding processes, we have documented here the process used for the development of an AUD codelist within primary care which can be used as a reference for future research.

## Introduction

Electronic Health Records (EHR) are a valuable resource for health research, as a source of data on medical diagnoses and treatments for a very large number of people, however their use comes with challenges. While increasingly large amounts of data collected during clinical consultations are available for health research, these data are not collected for research purposes and are subject to variation in coding practices among clinicians.^1^ This can lead to issues such as measurement error and high levels of missing data.

Alcohol-related harm has substantial implications both for the health of individuals and for society more broadly. Alcohol use was ranked as the seventh leading risk factor for premature mortality and disability globally in 2016 and the leading risk factor for risk-attributable disease burden in people aged 15–49 years.^2^ A recent study from the Institute of Alcohol Studies estimated the annual economic cost of alcohol-related harm in England is £27.44 billion.^3^

Alcohol Use Disorder (AUD) is an umbrella term that encompasses alcohol dependence and harmful use of alcohol. AUD can lead to a range of negative impacts including psychological, physical and social harms. People with AUD have substantially higher mortality compared with the general population with estimates ranging from two to four times higher risk.^4^ AUD is linked to health inequalities^5^,^6^ and people with AUD experience a high burden of comorbidities, including other mental health comorbidities.^7^

Previous research has been conducted on the coding of alcohol screening^8^ and alcohol intake^9^ within UK primary care EHR data. This showed there were low levels of recording of alcohol use within primary care with only 50% of 1.8 million patients having a record related to alcohol use in the past 5 years in 2018.^9^ However the codes used for AUD have not yet been investigated in detail. Understanding the coding of AUD in primary care is important for improving population-based research for this patient group.

The Health Data Research UK (HDRUK) phenotype library is an open access repository for algorithms for identifying phenotypes from EHR data.^10^ Researchers are encouraged to publish their existing codelists there to improve research transparency and sharing of codelists. In searching the HDRUK phenotype library for phenotypes including the term “alcohol”, six potential alcohol use disorder codelists were identified labelled as follows: alcohol dependence (n=1); alcohol abuse (n=2); alcohol problems (n=1); clinically significant alcohol misuse (n= 1) and alcohol (not specified) (n=1). While many of these codelists had similarities in the codes included, all had different numbers of codes, ranging from 40 to 110.^11^ Given research studies have differing aims and objectives there are likely valid reasons why different studies would include or exclude different codes. It is important to document the reasons behind the development of a particular codelist. To our knowledge, no validation studies investigating the sensitivity and specificity of AUD coding within EHR data have been conducted.

The Clinical Practice Research Datalink (CPRD) primary care database consists of anonymised EHR data from general practices (GP) in the United Kingdom. CPRD includes data collected through two main and different software systems used in primary care: Vision^®^ and Egton Medical Information System Ltd. (EMIS web^®^). Because of differences in structure and coding between the systems, these are provided as separate datasets: CPRD GOLD for Vision^®^ data^12^ and CPRD Aurum for EMIS data.^13^ In recent years, the number of general practices within CPRD using EMIS software has increased and the number using Vision^®^ has decreased. This means that for future research, more research resources which can be used in CPRD Aurum will be essential to make the best use of the available data. All existing AUD codelists identified through a literature search or HDRUK phenotype library were developed for use within CPRD GOLD. To the best of our knowledge, a validated AUD codelist for the CPRD Aurum has not yet been developed. As additional codes are added to the CPRD Aurum over time, it is essential to document the codelist creation process for the repeatability of methods when updating codelists.

Our objectives were 1) to develop a publicly available AUD codelist for use in the CPRD Aurum database, 2) document the methodology used in the codelist development process, and 3) discuss the interpretability of AUD coding in primary care. In doing so, we aimed to ensure that this process is repeatable and transparent for future research.

## Material and Methods

A search strategy was developed to identify codes related to alcohol use within CPRD Aurum. Codes (known as medcodes) for observations within CPRD Aurum are a combination of SNOMED CT,^14^ Read codes^15^ and local EMIS Web ^®^ codes.^13^

The AUD codelist was developed following publicly available guidelines for creating a SNOMED CT codelist:^16^

1. The CPRD code browser (February 2022) was used to search for words related to alcohol, using the search strategy developed previously by Mansfield et al.^9^
2. Exclusion criteria were added to remove codes unrelated to alcohol; however, this did not result in exclusion of any terms.
3. A search was performed using SNOMED concept IDs of the remaining codes to include synonyms. While CPRD Aurum uses unique IDs (medcodeids) for each code, these codes also have a SNOMED Concept ID, which is an ID for a single concept which may have multiple concepts associated with it. For example, SNOMED ConceptID 66590003 for alcohol dependence is associated with multiple medcodeids.
4. Codelists from two older studies developed using the same search terms^9^,^17^ were merged with this list.

The search terms and code used to develop the initial sample of codes are available on Github (https://github.com/NHLI-Respiratory-Epi/Alcohol_use_disorder_codelist).

### Alcohol Use Disorder Definition

AUD was used as an “umbrella term” to cover ICD-10 diagnoses of alcohol dependence syndrome and harmful alcohol use as defined in Chapter F10.1-F10.9 Mental and Behavioural disorders due to Alcohol.^18^ Alcohol dependence syndrome within ICD-10 is defined as

A cluster of behavioural, cognitive, and physiological phenomena that develop after repeated alcohol use and that typically include a strong desire to use alcohol, difficulties in controlling its use, persisting in its use despite harmful consequences, a higher priority given to alcohol use than to other activities and obligations, increased tolerance, and sometimes a physical withdrawal state.

Harmful alcohol use is defined as “A pattern of alcohol use causing damage to health”.^18^

In line with clinical diagnoses, codes were considered to indicate probable AUD if they included 1) a clinical diagnosis term indicating alcohol use disorder, 2) evidence of withdrawal from alcohol, or 3) chronic alcohol-related harm. Chronic alcohol-related harm was defined as any health-related harm attributed directly to alcohol. This included both harm to physical health for example alcohol-related liver disease and harm to mental health for example alcohol-related dementia. Acute harms which could be caused by consumption of alcohol on one drinking occasion for example alcohol poisoning or injuries were not included as part of the definition of alcohol use disorder but were classified separately within the codelist.

Additionally, codes which did not specifically indicate AUD but could be considered to contain useful information about a person’s alcohol use were categorised as i)codes indicating treatment or management related to alcohol use; ii) AUD-related (not specific enough to provide a clinical diagnosis of AUD but showing some evidence for this); iii) alcohol status (code could be used to indicate whether someone drinks alcohol or not, but not necessarily providing evidence of AUD); iv) indicator of alcohol screening from an established alcohol screening tool for primary care (Alcohol Use Disorders Identification Test (AUDIT), AUDIT-C, or FAST);^19^,^20^ or v) another screening tool and vii) acute alcohol-related harm (eg acute intoxication, alcohol poisoning, or injury).

### Clinical Review

An initial review of all codes identified was conducted by the first author (SC), who is medically qualified but not practising. All codes were classified according to the classification system listed in Table 1. At this stage, SC removed codes recorded less than 10 times and codes which were not related to alcohol use. Codes recorded less than 10 times were removed 1) to limit the possibility of identification of individuals through small numbers and 2) remove infrequently used codes which may represent coding specificities of certain general practitioners (GPs) or practices.

**Table 1:** Categorisation of Codes

	Code category	Final number of codes
Used to define AUD	AUD disorder diagnosis	38
	Withdrawal	14
	Alcohol-related harm –physical health chronic harm	43
	Alcohol-related harm – mental health chronic harm	44
Not used to define AUD but considered contained useful information about alcohol use	Alcohol management	60
	AUD-related	0
	Alcohol status	93
	AUDIT score; AUDIT-C; FAST score	34
	Other screening	20
	Acute alcohol harm	48
Codes which could not be used	Code not related to alcohol	393
	Code use not interpretable	162
	Codes used less than 10 times	583

The remaining codes were split into blocks of 100 and each block reviewed by one of the co-authors (AM, RP, SG, TB, and ALN), all of whom are practising GPs in primary care within England who performed a second independent blind coding. After the first 100 codes were categorised, the coding between 1^st^ coder (SC) and 2^nd^ coder was reviewed for differences, and 1^st^ coder met either individually or with up to two of the 2^nd^ coders to discuss the process and any disagreements before assigning another sample of codes.

After all codes had been independently coded, all cases in which there was disagreement between the first and second coders were discussed by at least three of the six coders, and consensus was reached on coding. The final codelist was shared with all co-authors who had the opportunity to challenge any of the codes assigned.

### Update of Codes from the Most Recent Code Browser (December 2023)

The CPRD code browser changes over time with the addition of additional code. To ensure that the codelist was up to date prior to publication, the search strategy was run again on the most recent version of the code browser (December 2023) and any additional codes not in the February 2022 code browser used more than 10 times were categorised independently by two of the original coders (SC, SG).

### Statistical Analysis

The Kappa statistic was used to investigate the reliability of the categorisation of alcohol codes overall and for AUD classified as binary (yes/no) between the first coder and each of the five second coders.

All analysis was conducted using Stata 17.

## Results

### Codelist Development

In total, 1532 codes were found from the initial search. After exclusion of codes which were unrelated to alcohol (n=393) and codes used less than 10 times ever (n=583), a total of 556 codes remained and were independently coded by GPs.

**Figure 1:**
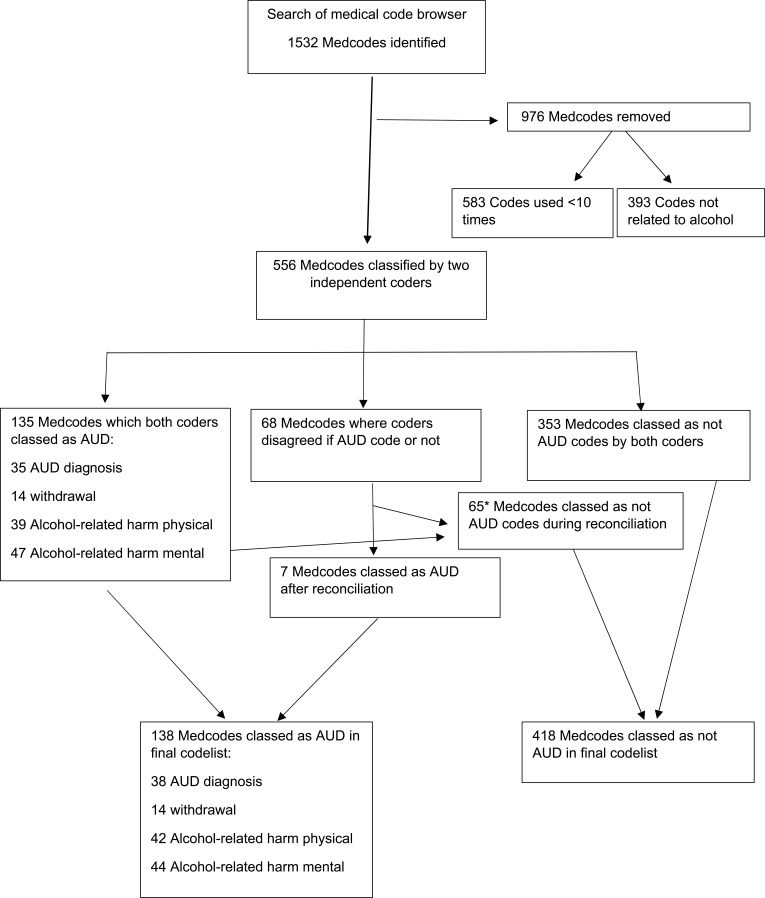
Process for deriving AUD Codelist.

**Notes**: *For consistency where codes were very similar the consensus code was applied to all codes even if both codes agreed in the first screen. 4 medcodeids classed as AUD by both coders initially were changed to non-AUD after reconciliation.

The number of codes categorised at each stage into the different categories is shown in Figure 1. After the independent coding process, there were 231 codes with disagreement between the coders. These were discussed and 98 codes changed from the original coding by the first coder. The final codelist included 137 codes classed as probable AUD: 38 codes including terms for clinical diagnosis of AUD, 14 indicating alcohol withdrawal plus 85 codes indicating chronic alcohol-related harm (41 physical health and 44 mental health). Following independent coding and consensus processes, no codes were classified as AUD-related. The final number of codes for each category is listed in Table 1.

The most frequently used code was “Alcohol dependence syndrome” (used 1,000,000 times) followed by “Alcoholism” (used 200,000 times), “Alcohol dependence syndrome NOS” (used 100,000 times) and “Alcoholic cirrhosis of liver” (used 100,000 times).

The level of reliability of categorisation of codes between the first and each of the second coders is shown in Table 2. Kappa for reliability between coders was moderate for both AUD (0.72) and across all categories (0.62), with substantial variability in the range between coders (AUD: 0.33–0.97; all categories 0.36–0.74).

**Table 2:** Reliability of Coding Between 1^st^ (Coder 1) and 2^nd^ Coder (Coders 2–6)

		All categories		AUD	
Coder	Number of codes reviewed	% Agreement	Kappa	% Agreement	Kappa
Coder 2	200	74.1	0.71	94.0	0.85
Coder 3	166	68.7	0.64	95.8	0.85
Coder 4	166	65.8	0.60	90.9	0.69
Coder 5	100	79.0	0.74	99.0	0.97
Coder 6	100	43.0	0.36	62.0	0.33
All codes	689*	67.1	0.62	89.6	0.72

Notes: *Some codes were coded by more than one person therefore the total number here is higher than the total number of codes.

### Consensus Discussion

There were three main discussion points in the consensus process:

1) Distinguishing between chronic physical and chronic mental health harms. A common reason for disagreement between coders was whether health harms were classed as physical or mental when the harm affected the brain and cognitive functioning, for example, alcohol-related dementia and amnestic syndromes. The decision made was to classify these as physical harms if a specific target organ was involved. Since our overarching definition of AUD included both physical and mental health harms, this decision did not substantively impact the final AUD codelist, but could affect future research if there was a need to separate physical and mental health harms.

2) How to classify codes which indicated some level of alcohol-related harm but were not specific with regard to ICD-10. For example, a commonly used term was “non-dependent alcohol abuse”. Variations of this term were used in total 154,900 times. The meaning of this code was not clear to the coders involved and it was difficult to know how to treat it. Within the reconciliation process, the decision made was to take a more specific rather than sensitive approach and only include as AUD codes which we felt confident reflected ICD-10 criteria for diagnosis.

3) Harmful alcohol use: How best to treat this code led to considerable discussion in the reconciliation process. The code is consistent with ICD-10 criteria as harmful alcohol use is a diagnostic term (F10.2). However, there was concern that this code would not be used in this way in practice by GPs and may instead reflect concern about someone’s drinking, or indicating short-term use, without the intention to diagnose AUD. The final decision was that without evidence of a specific chronic health harm from alcohol, this code alone would not be sufficient for defining AUD.

### Update of Codelist Using December 2023 Code Browser

When the same search strategy was applied to a more recent version of the code browser, 166 additional alcohol-related codes were identified. Of these, 33 were used more than ten times. Two of the coders (SC and SG) reviewed these 33 codes independently and agreed on an additional 9 codes for AUD (1 for alcohol withdrawal and 8 for chronic alcohol-related harm). These additional 9 codes were added to the AUD codelist.

The final codelist for AUD is available on Github (https://github.com/NHLI-Respiratory-Epi/Alcohol_use_disorder_codelist) (Supplementary File)

## Discussion

Here we developed a recommended codelist for AUD research for use in CPRD Aurum. We found that a large number of codes related to alcohol use were used by GPs. The independent coding and reconciliation process revealed the difficulty of interpreting these codes, with only moderate agreement between the coders regarding what should be classified as AUD (kappa 0.72). After discussion of conflicting codes, the final list included codes we felt more certain indicated AUD, but that meant that less specific codes were disregarded. While we favoured specificity over sensitivity, depending on the purpose of the research, our approach could be adapted to include a broader range of less specific codes from the full list of alcohol codes identified through a review of the codes included within the codelist in the other categories (alcohol status, alcohol management and acute alcohol-related harm).

Although we have considered AUD here, our methodological approach can be applied to create code lists for other conditions. In sharing our approach, we are aligned with increasing moves to improve transparency and the sharing of analytical codes and codelists for health research, such as through sharing codelists with the HDRUK phenotype library or specific disease-focused projects such as BREATHE^21^ or The Diabetes Data Science Catalyst.^22^ Initiatives to join up researchers using these data to share experience and knowledge are vital to make the best use of these datasets and to improve the quality of research.

## Limitations

EHR research relies primarily on the use of codelists for identifying diagnoses of interest for the researcher (as an exposure, outcome or a confounder). A person is then considered to have or not have this diagnosis. Although this practice is a practical necessity for using these complex datasets, it may not reflect the diagnostic uncertainty on the part of the clinician entering the code in clinical practice. Furthermore, the use of a single code does not capture how a patient’s clinical history changes or develops over time. The timeframe used to define a long-term condition has been shown to have a strong impact on the prevalence of multi-morbidity.^23^ While we attempted to define a list of codes in use within primary care which would indicate that someone has ever had an AUD, it is important to emphasise that this does not capture much of the complexity involved in understanding AUD or its progression across the life course.

It is also important to note that the codes used in general practice change over time, as can be seen by the addition of 166 new codes related to alcohol in the 2 year window between the start and end of this process (February 2022 code browser versus December 2023 code browser). Additionally, this process included codes that were historically recorded in primary care records. Some of the terms included are out of date with current diagnostic terminology, and in some cases, their use is not recommended because of the potential for stigma.^24^,^25^ For example, “Alcoholism” is less favoured as a term in clinical practice^25^ however, it was one of the most commonly used codes used over 200,000 times, reflecting changes in diagnostic terminology and understanding of AUD over time. While here we have not removed codes using stigmatising language from our codelist as for research purposes as this would lead to bias in identifying people with AUD further back in time, this process high lights the importance of review of medical codes in use within current clinical practice to ensure potentially stigmatising language is removed from available codes going forward.

While we aimed to obtain consensus on the use of codes using an independent coding process and several coders, the decision-making process on reaching consensus involved an element of subjectivity. However, a strength of our approach is that we captured a range of views from practising GPs. Our method describing the process of searching for relevant codes then agreeing consistent categories with a group of clinicians can be applied to other long term conditions. Here we have developed a codelist which could be used for any future research in CPRD Aurum which includes AUD either as a risk factor, outcome or a co-variate.

## Conclusions and Future Work

Here, we share the development process for a codelist for AUD to improve the quality of population-based research for this patient group within UK primary care. Further work formally validating this codelist against gold standard clinical review is required to understand GPs’ coding processes. Qualitative work is required to provide a better understanding of how different codes are used. Future work should also include a focus on ensuring the use of non-stigmatising language within medical codes.

## Data Availability

Data are available on request from the CPRD. Their provision requires the purchase of a license, and this license does not permit the authors to make them publicly available to all. Licenses are available from the CPRD (http://www.cprd.com): The Clinical Practice Research Datalink Group, The Medicines and Healthcare products Regulatory Agency, 10 South Colonnade, Canary Wharf, London E14 4PU. The Alcohol Use Disorder codelist generated as part of this is available on GitHub (https://github.com/NHLI-Respiratory-Epi/Alcohol_use_disorder_codelist)

## References

[1] Shemtob L, Beaney T, Norton J, Majeed A. How can we improve the quality of data collected in general practice? *BMJ*. 2023;380:e071950. doi:10.1136/bmj-2022-071950 36921932

[2] Griswold MG, Fullman N, Hawley C, et al. Alcohol use and burden for 195 countries and territories, 1990–2016: a systematic analysis for the Global Burden of Disease Study 2016. *Lancet*. 2018;392(10152):1015–1035. doi:10.1016/S0140-6736(18)31310-2 30146330 PMC6148333

[3] Institute of Alcohol Studies. Economy Availabe from: https://www.ias.org.uk/factsheet/economy/#htoc-reports2024. Accessed september 20, 2024.

[4] Roerecke M, Rehm J. Alcohol use disorders and mortality: a systematic review and meta-analysis. *Addiction*. 2013;108(9):1562–1578. doi:10.1111/add.12231 23627868

[5] Probst C, Kilian C, Sanchez S, Lange S, Rehm J. The role of alcohol use and drinking patterns in socioeconomic inequalities in mortality: a systematic review. *Lancet Public Heal*. 2020;5(6):e324–e32. doi:10.1016/S2468-2667(20)30052-9 32504585

[6] Katikireddi SV, Whitley E, Lewsey J, Gray L, Leyland AH. Socioeconomic status as an effect modifier of alcohol consumption and harm: analysis of linked cohort data. *Lancet Public Heal*. 2017;2(6):e267–e76. doi:10.1016/S2468-2667(17)30078-6 PMC5463030 28626829

[7] Lai HM, Cleary M, Sitharthan T, Hunt GE. Prevalence of comorbid substance use, anxiety and mood disorders in epidemiological surveys, 1990-2014: a systematic review and meta-analysis. *Drug Alcohol Depend*. 2015;154:1–13. doi:10.1016/j.drugalcdep.2015.05.031 26072219

[8] O’Donnell A, Haighton C, Chappel D, Shevills C, Kaner E. Impact of financial incentives on alcohol intervention delivery in primary care: a mixed-methods study. *BMC Family Practice*. 2016;17(1):165. doi:10.1186/s12875-016-0561-5 27887577 PMC5124277

[9] Mansfield K, Crellin E, Denholm R, et al. Completeness and validity of alcohol recording in general practice within the UK: a cross-sectional study. *BMJ Open*. 2019;9(11):e031537. doi:10.1136/bmjopen-2019-031537 PMC6887039 31772094

[10] HDR UK CALIBER Phenotype Library. Availabe from: https://portal.caliberresearch.org/2024. Accessed september 20, 2024.

[11] HDR UK CALIBER Phenotype Library. Available from: https://portal.caliberresearch.org/. Accessed september 20, 2024.

[12] Herrett E, Gallagher AM, Bhaskaran K, et al. Data Resource Profile: clinical Practice Research Datalink (CPRD). *Int J Epidemiol*. 2015;44(3):827–836. doi:10.1093/ije/dyv098 26050254 PMC4521131

[13] Wolf A, Dedman D, Campbell J, et al. Data resource profile: clinical Practice Research Datalink (CPRD) Aurum. *Int J Epidemiol*. 2019;48(6):1740–g. doi:10.1093/ije/dyz034 30859197 PMC6929522

[14] NHS Digital. SNOMED CT. Availabe from: https://digital.nhs.uk/services/terminology-and-classifications/snomed-ct2023. Accessed september 20, 2024.

[15] NHS Digital. Read Codes. Availabe from: https://digital.nhs.uk/services/terminology-and-classifications/read-codes2023. Accessed september 20, 2024.

[16] Rolova G, Gavurova B, Petruzelka B. Health Literacy, Self-Perceived Health, and Substance Use Behavior among Young People with Alcohol and Substance Use Disorders. *Int J Environ Res Public Health*. 2021;18(8):4337. doi:10.3390/ijerph18084337 33921885 PMC8073264

[17] Mansfield KE, Mathur R, Tazare J, et al. Indirect acute effects of the COVID-19 pandemic on physical and mental health in the UK: a population-based study. *Lancet Digital Health*. 2021;3(4):e217–e30. doi:10.1016/S2589-7500(21)00017-0 33612430 PMC7985613

[18] World Health Organization. *International Statistical Classification of Diseases and Related Health Problems*. 10th revision, Fifth edition, 2016. Geneva: World Health Organization; 2015.

[19] Saunders JB, Aasland OG, Babor TF, La Fuente JR D, Grant M. Development of the Alcohol Use Disorders Identification Test (AUDIT): WHO Collaborative Project on Early Detection of Persons with Harmful Alcohol Consumption-II. *Addiction*. 1993;88(6):791–804. doi:10.1111/j.1360-0443.1993.tb02093.x 8329970

[20] Hodgson R, Alwyn T, John B, Thom B, Smith A. THE FAST ALCOHOL SCREENING TEST. *Alcohol Alcohol*. 2002;37(1):61–66. doi:10.1093/alcalc/37.1.61 11825859

[21] Hatam S, Scully ST, Cook S, et al. Harmonised Approach to Curating Research-Ready Datasets for Asthma, Chronic Obstructive Pulmonary Disease (COPD) and Interstitial Lung Disease (ILD) in England, Wales and Scotland Using Clinical Practice Research Datalink (CPRD). *Clin Epidemiol*. 2024;16:235–247. doi:10.2147/CLEP.S437937 38595770 PMC11002787

[22] Health Data Research UK. Diabetes Data Science Catalyst. Availabe from: https://www.hdruk.ac.uk/helping-with-health-data/bhf-data-science-centre/diabetes-data-science-catalyst/2024. Accessed september 20, 2024.

[23] Beaney T, Clarke J, Woodcock T, Majeed A, Barahona M, Aylin P. Effect of timeframes to define long term conditions and sociodemographic factors on prevalence of multimorbidity using disease code frequency in primary care electronic health records: retrospective study. *BMJ Med*. 2024;3(1):e000474. doi:10.1136/bmjmed-2022-000474 PMC10868275 38361663

[24] Kelly JF, Saitz R, Language WS. Substance Use Disorders, and Policy: the Need to Reach Consensus on an “Addiction-ary”. *Alcohol Treat Quarter*. 2016;34(1):116–123. doi:10.1080/07347324.2016.1113103

[25] National Institute of Alcohol Abuse and Alcoholism. When It Comes to Reducing Alcohol-Related Stigma, Words Matter. Available from: https://www.niaaa.nih.gov/alcohols-effects-health/reducing-alcohol-related-stigma[. Accessed september 20, 2024.

